# Fungicide-Driven Evolution and Molecular Basis of Multidrug Resistance in Field Populations of the Grey Mould Fungus *Botrytis cinerea*

**DOI:** 10.1371/journal.ppat.1000696

**Published:** 2009-12-18

**Authors:** Matthias Kretschmer, Michaela Leroch, Andreas Mosbach, Anne-Sophie Walker, Sabine Fillinger, Dennis Mernke, Henk-Jan Schoonbeek, Jean-Marc Pradier, Pierre Leroux, Maarten A. De Waard, Matthias Hahn

**Affiliations:** 1 Department of Biology, University of Kaiserslautern, Kaiserslautern, Germany; 2 UMR1290 BIOGER-CPP, INRA-AgroParisTech, Thiverval-Grignon, France; 3 Institute of Plant Science, University of Fribourg, Fribourg, Switzerland; 4 Laboratory of Phytopathology, Wageningen University, Wageningen, The Netherlands; University of Melbourne, Australia

## Abstract

The grey mould fungus *Botrytis cinerea* causes losses of commercially important fruits, vegetables and ornamentals worldwide. Fungicide treatments are effective for disease control, but bear the risk of resistance development. The major resistance mechanism in fungi is target protein modification resulting in reduced drug binding. Multiple drug resistance (MDR) caused by increased efflux activity is common in human pathogenic microbes, but rarely described for plant pathogens. Annual monitoring for fungicide resistance in field isolates from fungicide-treated vineyards in France and Germany revealed a rapidly increasing appearance of *B. cinerea* field populations with three distinct MDR phenotypes. All MDR strains showed increased fungicide efflux activity and overexpression of efflux transporter genes. Similar to clinical MDR isolates of *Candida* yeasts that are due to transcription factor mutations, all MDR1 strains were shown to harbor activating mutations in a transcription factor (Mrr1) that controls the gene encoding ABC transporter AtrB. MDR2 strains had undergone a unique rearrangement in the promoter region of the major facilitator superfamily transporter gene *mfsM2*, induced by insertion of a retrotransposon-derived sequence. MDR2 strains carrying the same rearranged *mfsM2* allele have probably migrated from French to German wine-growing regions. The roles of *atrB*, *mrr1* and *mfsM2* were proven by the phenotypes of knock-out and overexpression mutants. As confirmed by sexual crosses, combinations of *mrr1* and *mfsM2* mutations lead to MDR3 strains with higher broad-spectrum resistance. An MDR3 strain was shown in field experiments to be selected against sensitive strains by fungicide treatments. Our data document for the first time the rising prevalence, spread and molecular basis of MDR populations in a major plant pathogen in agricultural environments. These populations will increase the risk of grey mould rot and hamper the effectiveness of current strategies for fungicide resistance management.

## Author Summary

Bacterial and fungal pathogens cause diseases in humans and plants alike. Antibiotics and fungicides are used for disease control, but the microbes are able to adapt quickly to these drugs by mutation. Multiple drug resistance (MDR) is well investigated in human pathogens and causes increasing problems with antibiotic therapy. Driven by the continuous use of fungicides in commercial vineyards, three types of rapidly increasing multidrug resistant populations of the grey mould fungus *Botrytis cinerea* have appeared in French vineyards since the mid 1990s. Using a combination of physiological, molecular and genetic techniques, we demonstrate that these MDR phenotypes are correlated with increased drug efflux activity and overexpression of two efflux transporters. Just two types of mutations, one in a regulatory protein that controls drug efflux, and the other in the gene for an efflux transporter itself, are sufficient to explain the three MDR phenotypes. We also provide evidence that a subpopulation of the French MDR strains has migrated eastward into German wine-growing regions. We anticipate that by continuous selection of multi-resistant strains, chemical control of grey mould in the field will become increasingly difficult.

## Introduction

Synthetic fungicides are used worldwide that provide protection of major crops from destruction by fungal plant pathogens [1]. As a result of repeated fungicide treatments, however, resistant strains of the pathogens are being selected [2]. Different resistance mechanisms have been reported that reduce fungicide effectiveness in field and greenhouse environments. Mutations leading to changes in the target proteins that are still functional but less sensitive to the drugs are most common in plant pathogenic fungi. For example, rapid accumulation of mutations in the gene encoding ß-tubulin have been observed in a variety of plant pathogens after introduction of the benzimidazole fungicides, leading to resistance against these fungicides [3]. Other mechanisms, such as overexpression of the gene encoding the target site or increased fungicide metabolism have also been described [3]–[6].

Multidrug resistance (MDR), an important resistance mechanism in human pathogenic microbes and cancer cells, has often been correlated with the activity of energy dependent plasma membrane efflux transporters with low substrate specificity. Mutations leading to overexpression of individual transporters can result in increased export and thereby reduced sensitivity to a variety of drug molecules [7]–[9]. In fungi, the major types of drug efflux proteins are ATP binding cassette (ABC) and major facilitator superfamily (MFS) transporters [10]–[12]. Constitutive overexpression of the ABC transporters CDR1 and CDR2, or the MFS transporter MDR1 has been observed in *Candida* spp. with MDR phenotypes that have been selected by prolonged fluconazole treatments in humans [13].

In filamentous fungi, the role of ABC and MFS transporters in the efflux of natural and synthetic toxicants is well known [14]. For example, analysis of knock-out mutants revealed that the ABC transporter AtrB from *Aspergillus nidulans* and its orthologue AtrB from *Botrytis cinerea* transport a wide variety of fungicides as well as toxins of plant and microbial origins [14]–[16]. Similarly, the MFS transporter Mfs1 of *B. cinerea* was found to mediate efflux of several fungicides as well as plant derived and microbial toxins [17]. Several efflux transporter genes have been shown to be rapidly induced by fungicides or natural toxins, such as *Mycosphaerella graminicola Atr1* and *Atr2*, or *B. cinerea atrD* and *atrB* [16],[18]. Some ABC transporters have been shown to be involved in plant pathogenesis [19]–[22]. This is probably mainly due to the export of plant defence compounds, for example the *Arabidopsis* phytoalexin camalexin in the case of *B. cinerea* AtrB [22]. For the ABC1 transporter of *Magnaporthe grisea*, evidence was provided that it is required for tolerance to oxidative stress during appressorial penetration [19].

Despite some reports of MDR phenotypes in laboratory mutants of *B. cinerea* [16],[18] and field strains of *Penicillium digitatum* and *Mycosphaerella graminicola* [23],[24], a significant role of MDR in agricultural environments has not yet been described for plant pathogens. However, a long term monitoring for fungicide resistance of *B. cinerea* initiated in French wine-growing regions has revealed, in addition to drug specific resistance mechanisms, the appearance of strains with cross resistance to chemically unrelated fungicides in the Champagne [4],[25],[26]. In this report, we have investigated these strains in further detail and describe the increasing prevalence of three different MDR populations in commercial vineyards. We show that their phenotypes are caused by mutations leading to overexpression of efflux transporters, and present evidence for long-distance migration of MDR strains from France to German wine-growing regions.

## Results

### Increasing occurrence of *B. cinerea* MDR strains in commercial vineyards

**Figure 1:**
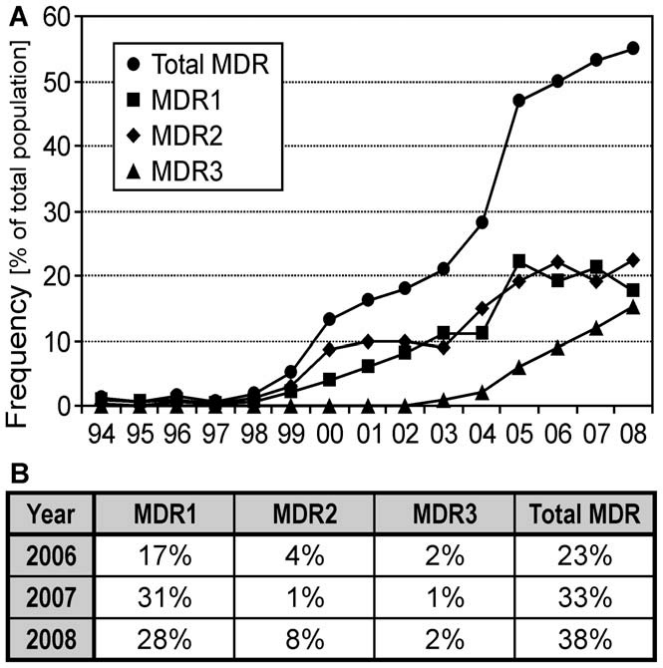
Isolation frequencies of *B. cinerea* MDR strains from French and German wine-growing regions. (A) Appearance of MDR strains in the Champagne. While MDR1 and MDR2 strains, named initially AniR2 and AniR3, respectively, were first detected in 1994 [4],[25], MDR3 strains have been observed since 2001. (B) Frequency of MDR strains in the German Wine Road region.

In 1994, strains with two different MDR phenotypes, formerly designated AniR2 (here MDR1) and AniR3 (here MDR2), because of their reduced sensitivity to anilinopyrimidine fungicides, have been identified for the first time in the Champagne (Fig. 1A) [26]. A third MDR phenotype (MDR3) was first detected in 2001. Since then, the frequency of MDR strains in the Champagne steadily increased until 2008, when the three MDR phenotypes together represented 55% of the total population (Fig. 1A). In vineyards of the German Wine Road region, a similar survey of *B. cinerea* isolates for fungicide sensitivity was performed for three years. Between 2006 and 2008, increasing MDR populations were also observed, but in contrast to the Champagne the MDR1 phenotype was clearly dominating (Fig. 1B). As previously described [26], MDR1 and MDR2 strains had overlapping but distinct profiles of increased tolerance to a number of different classes of fungicides and other drugs (Table 1). Although the levels of tolerance observed were not as high as specific resistance mechanisms (e.g. target site mutations), they were clearly genetically based and heritable and therefore called resistance throughout this paper. While MDR1 strains showed considerable resistance levels mainly towards fludioxonil, cyprodinil and tolnaftate, MDR2 strains were characterized by increased resistance to fenhexamid, tolnaftate, cycloheximide and cyprodinil. MDR3 strains showed the highest levels and broadest spectrum of resistance against most fungicides tested (Table 1).

**Table 1:** Classification of *B. cinerea* MDR strains by their drug sensitivities.

	Fludioxonil	Fenhexamid	Cyprodinil	Carbendazim	Boscalid	Iprodione	Tebucoazole	Bitertanol	Tolnaftate^1^	Cycloheximide
**Sensitive strains (EC50: mg/l)**	0.03±0.01	0.05±0.01	0.006±0.001	0.04±0.01	0.08±0.01	1.03±0.08	0.66±0.31	2.6±0.65	0.65±0.08	3.2±0.68
	**Resistance factors (x fold of sensitive)**									
**MDR1 strains**	8.1x±1.3**	1.6x±0.5**^n.s.^**	18.2x±4.5*	2.8x±0.2***	1.6x±0.2**^n.s.^**	1.4x±0.3**^n.s.^**	0.7x±0.4**^n.s.^**	1.0x±0.4**^n.s.^**	20.4x±2.3***	0.7x±0.1**^n.s.^**
**MDR2 strains**	2.6x±0.3***	9.8x±0.6***	6.2x±2.8**	1.1x±0.1**^n.s.^**	2.0x±0.8*	5.4x±1.0***	1.8x±0.2*	1.7x±0.2*	>25x***	13.7x±1.5**
**MDR3 strains**	11.4x±1.9**	14.7x±3.2**	25.7x±5.2**	3.1x±0.5**	3.5x±0.9*	6.4x±1.0**	2.3x±0.6*	1.6x±0.1*	>25x***	14.1x±0.3***

Mean values from three French and three German isolates each of sensitive strains, MDR1 strains, MDR2 strains, and MDR3 strains are shown. For sensitive strains, EC_50_ values, and for MDR strains, resistance factors relative to the corresponding values of sensitive strains are shown. ^1^Due to limited solubility of tolnaftate, accurate values above 25-fold could not be determined. Significant differences to mean values of sensitive strains: n.s.: Not significant; * p<0.05; ** p<0.01; *** p<0.001.

### MDR phenotypes show increased efflux transporter activity and expression

**Figure 2:**
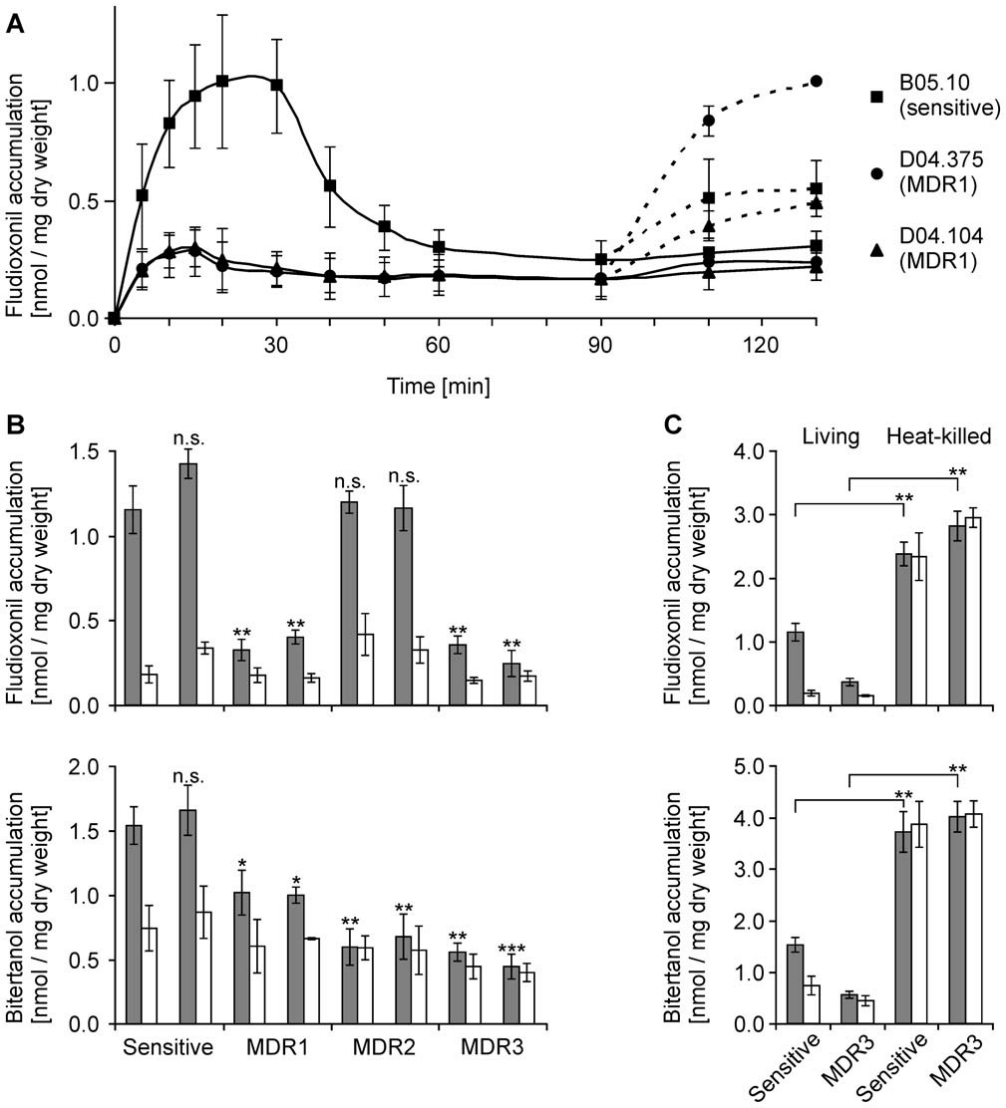
Differential fungicide accumulation by *B. cinerea* sensitive and MDR strains. (A) Kinetics of fludioxonil (^14^C-labeled) accumulation by germinated spores of sensitive strain B05.10 (square) and of MDR1 strains D04.375 (circle) and D04.104 (triangle). Addition of carbonyl cyanide *m*-chlorophenylhydrazone (CCCP, 10 µM) after 90 min led to a net influx of the fungicide into the cells (dashed lines). (B) Accumulation of ^14^C-fludioxonil and ^14^C-bitertanol by sensitive and MDR strains. Samples were taken 10 min (shaded bars) and 60 min (white bars) after addition of labeled fungicide. The following strains were analyzed (from left to right): B05.10, D06.6-15 (sensitive); D06.5-16, D04.375 (MDR1); D06.2-6, D06.6-5 (MDR2); D06.7-33, D06.7-39 (MDR3). Significant differences of values (10 min) to those of sensitive strain B05.10 are indicated: n.s.: not significant; * p<0.05; ** p<0.01; *** p<0.001. (C) Control experiments demonstrating large differences in ^14^C-fungicide uptake between living and heat-killed germlings of sensitive (B05.10) and MDR3 (D06.7-33) strains.

**Figure 3:**
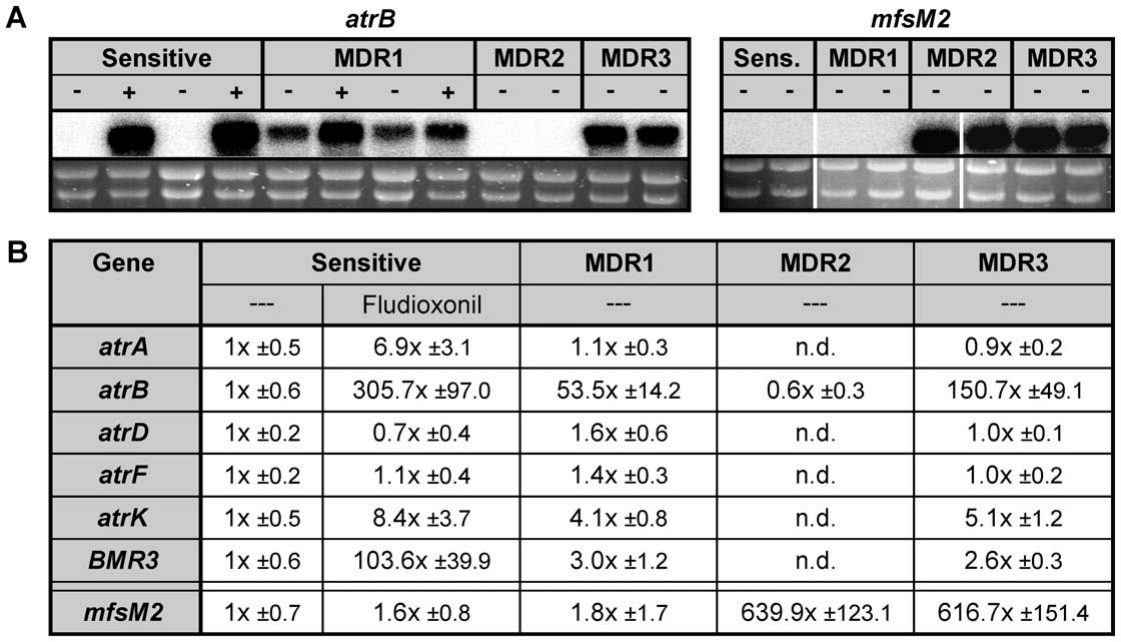
*B. cinerea* MDR strains show constitutive overexpression of efflux transporter genes. (A) Expression analysis by Northern hybridization of *atrB* and *mfsM2* in *B. cinerea* germlings. For hybridization with *atrB*, RNA of the following strains was loaded (from left to right): B05.10, D06.6-15 (sensitive, two lanes each); D06.5-16, D06.7-27 (MDR1, two lanes each); D06.6-5, D06.2-6 (MDR2); D06.7-39, D06.7-33 (MDR3). Below the hybridization signals, the corresponding RNA samples after agarose electrophoresis and ethidium bromide staining are shown as loading controls. ---: no treatment; +: 30 min treatment with 1 mg/l fludioxonil. For hybridization with *mfsM2*, RNAs of non-treated germlings were loaded in the same order as for *atrB*. (B) Expression analysis by quantitative RT-PCR of efflux transporter genes in sensitive and MDR strains. Values indicate fold-increases in expression levels, relative to the levels in sensitive strains without fludioxonil treatment (---). Mean values are shown from three strains each with sensitive, MDR1, MDR2, and MDR3 phenotypes. n.d.: Not determined.

MDR phenotypes in fungi are usually correlated with increased drug efflux [8]. When the *B. cinerea* MDR strains were tested, they showed indeed lower fungicide accumulation than sensitive strains, indicating increased efflux activity. As previously reported [16], sensitive strains show a transient accumulation of ^14^C-fludioxonil, followed by efflux of the drug after approximately 30 min, due to activation of efflux transporters (Fig. 2A). In contrast, two MDR1 strains showed only low initial fludioxonil accumulation. After addition of the uncoupler CCCP, rapid influx was observed for all strains indicating the presence of energy-dependent efflux systems (Fig. 2A) [16]. Similarly, MDR3 strains accumulated little fludioxonil, while MDR2 strains behaved similar to sensitive strains (Fig. 2B). These data indicated the presence of a constitutive efflux system in MDR1 and MDR3 strains. With ^14^C-bitertanol, all MDR strains showed reduced initial accumulation levels compared to sensitive strains, although this effect was less pronounced in MDR1 than in MDR2 and MDR3 strains (Fig. 2B). In accordance with these data, lower accumulation of tebuconazole and triadimenol by a *B. cinerea* strain with MDR2 phenotype has been described previously [25]. The specificity of the uptake experiments was confirmed by experiments with heat-inactivated germlings, which displayed very high, non-transient fungicide accumulation (Fig. 2C). The phenotype of MDR1 strains, including the low initial fludioxonil accumulation, was similar to that of a *B. cinerea* laboratory mutant which showed overexpression of the ABC transporter AtrB [16]. AtrB and its orthologs in other filamentous fungi, including *Aspergillus nidulans* and *Penicillium digitatum*, is a conserved efflux pump that contributes tolerance to various fungicides and natural antifungal compounds [14]–[16],[21],[22],[27],[28]. Indeed, *atrB* was constitutively upregulated in MDR1 and MDR3 strains, but not in MDR2 strains, showing 50–150 fold overexpression relative to sensitive strains. As previously reported, high levels of *atrB* expression were observed in sensitive strains after 30 min treatment with fludioxonil (Fig. 3) [16]. Two other genes encoding ABC transporters, *atrK* and *BMR3*, were also upregulated in the absence of drug induction in MDR1 and MDR3 strains when compared to sensitive strains, but only 2.5–5 fold (Fig. 3B). To identify efflux transporters that are specifically upregulated in MDR2 strains, microarray hybridizations with *B. cinerea* whole genome chips were performed (data not shown). These experiments revealed that *mfsM2* (Major facilitator superfamily transporter involved in MDR2), which showed very weak expression in sensitive and MDR1 strains, was more than 600 fold overexpressed in MDR2 and MDR3 strains (Fig. 3).

### Overexpression of transporter genes is responsible for MDR phenotypes

**Figure 4:**
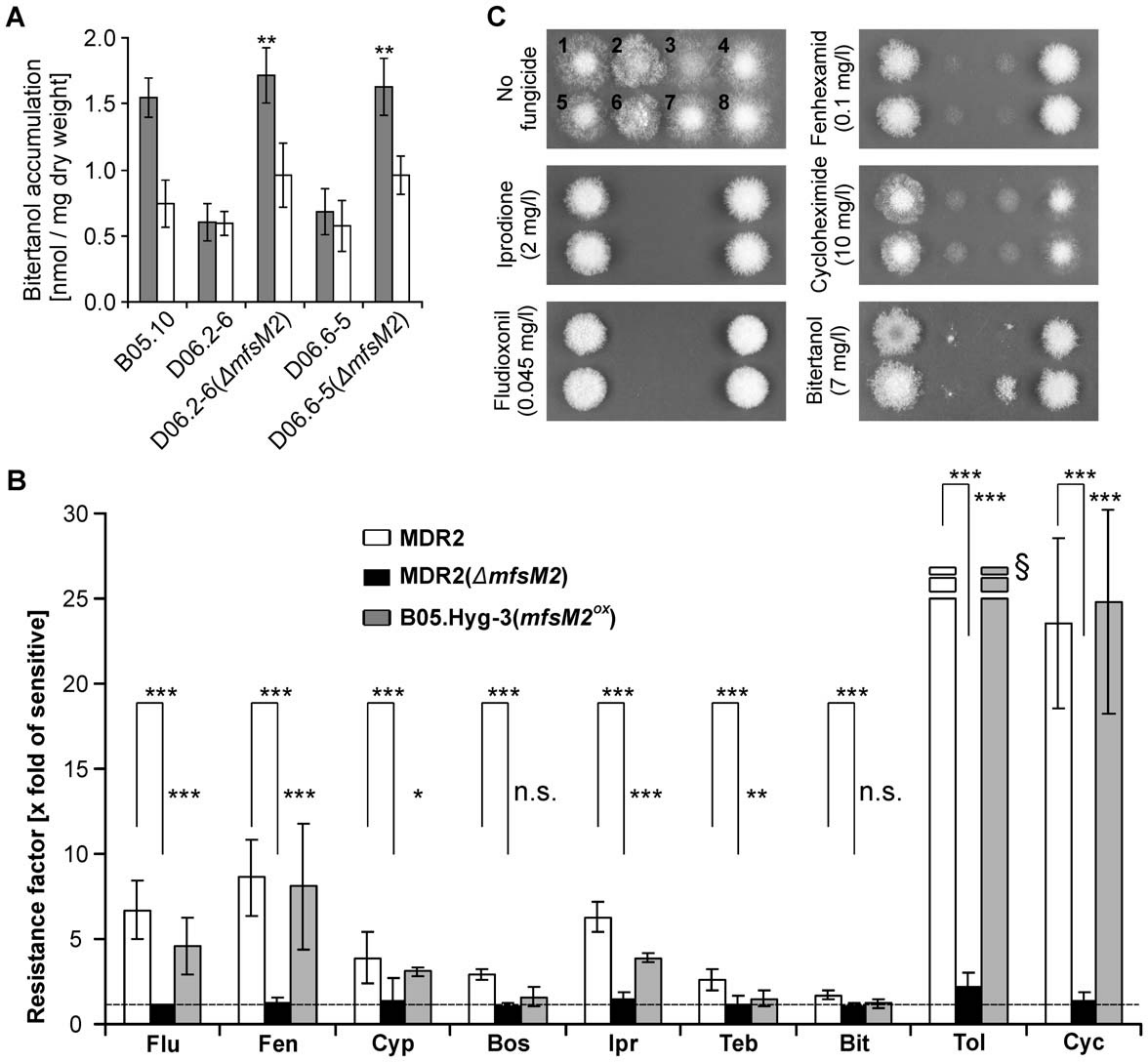
The MfsM2 efflux transporter controls MDR2. (A) Bitertanol (^14^C-labeled) accumulation after 10 min (shaded) and 60 min (white bars). Significant differences of values (10 min) of the mutants to those of their MDR2 parent strains are indicated: ** p<0.01. (B) Drug sensitivities. Mean values of resistance factors relative to B05.10 are shown, from two MDR2 strains (D06.2-6, D06.6-5; white bars), strains D06.2-6(*ΔmfsM2*) and D06.6-5(*ΔmfsM2*) (black bars), and from two transformants of strain B05.Hyg-3(*mfsM2^ox^*) (grey bars). Significant differences of corresponding values are indicated between MDR2 (*ΔmfsM2*) mutants and their parent strains, and between strain B05.Hyg-3(*mfsM2^ox^*) and strain B05.Hyg-3: n.s.: Not significant; * p<0.05; ** p<0.01; *** p<0.001. ^§^Due to limited solubility of tolnaftate, no accurate values above 25-fold could be determined. Drugs (abbreviated) are listed in the same order as in Table 1, except for the omission of carbendazim. (C) Drug sensitivity phenotypes on HA plates. 1: D06.2-6(MDR2); 2: D06.2-6(*ΔmfsM2*); 3: B05.10 (sensitive); 4: B05.Hyg-3(*mfsM2^ox^*)-4, 5: D06.6-5(MDR2); 6: D06.6-5(*ΔmfsM2*); 7: B05.Hyg-3 (sensitive); 8: B05.Hyg-3(*mfsM2^ox^*)-11. Strains with *mfsM2* mutations showed a slight growth difference to their parent strains. Pictures were taken 3 d.p.i., except for bitertanol (4 d.p.i.). Concentrations of drugs were adjusted to reveal clear differences between the strains which overexpress mfsM2 and those which do not.

To confirm a causal relationship between overexpression of efflux transporters and MDR phenotypes, *atrB* and *mfsM2* mutants were generated. As described further below, MDR1 strains with *atrB* deletions had lost the MDR phenotype. Two MDR2 strains with *mfsM2* deletions had lost increased efflux activity for ^14^C-bitertanol (Fig. 4A). Furthermore, the *mfsM2* deletion mutants had lost the reduced sensitivity to various fungicides, showing levels similar to sensitive strains (Fig. 4B, C). In contrast, when a sensitive strain was transformed with a construct providing constitutive overexpression of *mfsM2* (*mfsM2^ox^*), which led to 1481(±309)-fold upregulation of *mfsM2* relative to the parent strain, it acquired drug resistance levels similar to MDR2 strains (Fig. 4B, C). Overexpression of *atrB* and *mfsM2* is therefore necessary and probably sufficient to generate MDR1 and MDR2 phenotypes, respectively, in *B. cinerea* field strains. Apart from its role in MDR, we found only slight growth differences of the *mfsM2* deletion mutants. When tested for pathogenicity, the *mfsM2* deletion mutants showed no significant differences compared to their parent strains (data not shown).

Mutations leading to changes in gene expression are often located either in the promoters of these genes, or in regulatory genes. Sequencing of the *atrB* promoter regions from several sensitive, MDR1 and MDR3 strains did not reveal any MDR1-specific mutations (not shown). Since other ABC-transporter genes besides *atrB* were also found to be upregulated in MDR1 and MDR3 strains (Fig. 3B), we assumed that the MDR1 phenotype might have been generated by mutations in a regulatory gene. In order to locate the suspected MDR1-specific regulator gene, a map-based cloning approach was performed. When F1 progeny isolates of several crosses with MDR strains were analyzed, the segregation data confirmed that MDR1 and MDR2 phenotypes are determined by just one genetic locus each, and that strains with MDR3 phenotype can originate from recombination between MDR1 and MDR2 strains (Table S1) [26]. By identifying polymorphic molecular markers that cosegregate with MDR1 and MDR3 phenotypes in the F1 progeny, it was possible to localize and identify *mrr1* (multidrug resistance regulator 1) encoding a putative Zn(II)_2_Cys_6_ zinc cluster transcription factor (TF; Table S2) [29]. The genetic marker closest to *mrr1*, BC63-17, located 1.8 kb away from *mrr1*, showed 100 percent cosegregation with MDR1 phenotypes. Similarly, using crosses with MDR2 strains, a marker located just 1.6 kb away from the efflux transporter gene *mfsM2* was found completely cosegregate with MDR2 phenotypes, indicating that they are caused by mutations in *mfsM2* (Table S3).

### Activating mutations in the Mrr1 transcription factor lead to MDR1

**Figure 5:**
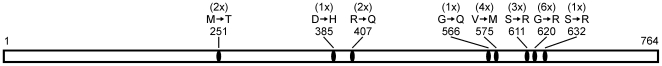
MDR1-related mutations in the Mrr1 transcription factor. Amino acid positions and exchanges found in MDR1 and MDR3 strains, and the observed frequencies of each mutation (in parentheses) are indicated. For a detailed list with Mrr1 sequences of individual strains, see Table S4.

**Figure 6:**
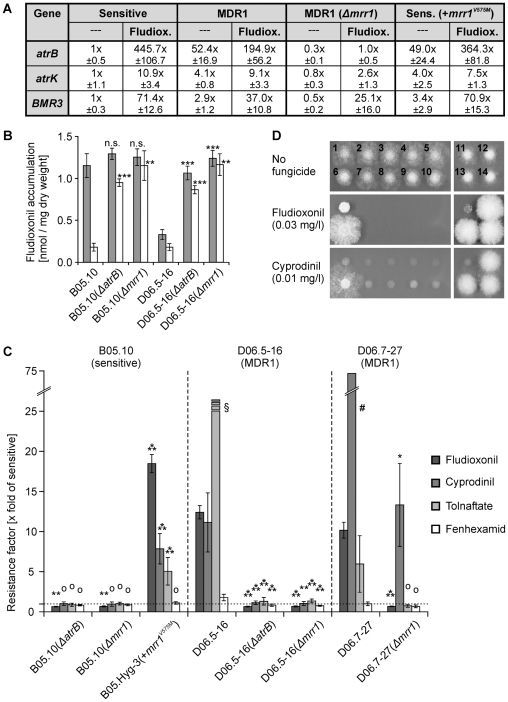
Mrr1 regulates MDR1 phenotypes via modulation of *atrB* expression. (A) Expression of ABC transporter genes in strains with different levels of *mrr1* expression. Mean values are shown from two sensitive strains (B05.10, B05.-Hyg3), two MDR1 strains (D06.5-16, D06.7-27), two MDR1 *mrr1* k.o. transformants (D06.5-16(*Δmrr1*)-5, -7), and two B05.Hyg-3 transformants expressing *mrr1^V575M^* (B05.Hyg-3(+*mrr1^V575M^*)-5, -6). (B) Fludioxonil (^14^C-labeled) accumulation by wild type strains and strains with mutations in *atrB* and *mrr1*, after 10 min (shaded) and 60 min (white bars). Significant differences between the values of the mutants and those of their parent strains are indicated separately for 10 min and 60 min values (n.s.: not significant; **: p<0.01; ***: p<0.001). (C) Drug sensitivities of *atrB* and *mrr1* mutants. For each mutant, mean values of two or three transformants were used to calculate resistance factors relative to sensitive strain B05.10 or (in case of B05.Hyg-3(+*mrr1^V575M^*) to strain B05.Hyg-3. ^#^MDR1 strain D06.7-27 showed higher resistance to cyprodinil (standard deviation = 12.0) and lower resistance to tolnaftate, compared to other MDR1 strains. Significantly different resistance values of the transformants relative to their parent strains are indicated (^O^ Not significant; * p<0.05; ** p<0.01; *** p<0.001). ^§^Due to limited solubility of tolnaftate, no accurate values above 25-fold could be determined. (D) Fungicide sensitivity phenotypes on agar plates. 1: B05.10 (sensitive); 2: B05.10(*ΔatrB*)-4; 3: B05.10(*ΔatrB*)-5; 4: B05.10(*Δmrr1*)-8; 5: B05.10(*Δmrr1*)-18; 6: D06.5-16 (MDR1); 7: D06.5-16(*ΔatrB*)-1; 8: D06.5-16(*ΔatrB*)-2; 9: D06.5-16(*Δmrr1*)-5; 10: D06.5-16(*Δmrr1*)-7; 11: B05.Hyg-3 (sensitive); 12: B05.Hyg-3(+*mrr1^V575M^*)-5; 13: B05.Hyg-3(+*mrr1^V575M^*)-6; 14: B05.Hyg-3(+*mrr1^V575M^*)-10. Top: HA, 2.5 d.p.i.; middle: HA, 0.03 mg/l fludioxonil, 4 d.p.i.; bottom: GB5 (glucose), 0.01 mg/l cyprodinil, 4 d.p.i..

Sequencing of *mrr1* from eight sensitive field strains revealed no or only silent nucleotide changes when compared to *mrr1* of the sequenced reference strains T4 and B05.10 (data not shown). In contrast, all MDR1 (n = 15) and MDR3 (n = 5) strains analyzed showed at least one point mutation leading to amino acid changes in Mrr1. In total, at least eight different MDR1-related mutations were identified (Fig. 5; Table S4). Their role in generation of the MDR1 phenotype was supported by the observation that five of these mutations had occurred in more than one MDR1 or MDR3 strain. To confirm that *mrr1* encodes the TF responsible for MDR1-related *atrB* overexpression, *mrr1* and *atrB* deletions were generated in MDR1 strains and a sensitive strain. Consistent with the expected phenotype of a regulatory mutant, *mrr1* mutants showed very low levels of *atrB* expression, not inducible by fludioxonil. Furthermore, they showed reduced expression of the ABC transporter genes *BMR3* and *atrK* that are also upregulated in MDR1 strains (Fig. 6A). Both the *mrr1* and *atrB* mutants of the MDR1 strain showed increased fludioxonil uptake, indicating loss of AtrB-mediated efflux activity (Fig. 6B). With regard to their drug sensitivity, the *mrr1* and *atrB* mutants of MDR1 strains had completely lost their MDR1 phenotypes and were slightly hypersensitive to fludioxonil, similar to previously described *atrB* mutants (Fig. 6C, D) [16]. The MDR1 strain D06.7-27 showed an unusually high cyprodinil resistance and a rather low tolnaftate resistance, compared to other MDR1 strains, for unknown reasons. In the D06.7-27(*Δmrr1*) mutant, the cyprodinil resistance was significantly reduced, but still higher than in sensitive strains (Fig. 6C). To confirm that single *mrr1* mutations are sufficient for generation of the MDR1 phenotype, a sensitive strain was transformed with the *mrr1^V575M^* allele from MDR1 strain D06.7-27. The transformants, expressing both wild type *mrr1* and *mrr1* ^V575M^, showed constitutive upregulation of *atrB* and, to a lower extent, *atrK* and *BMR3* (Fig. 6A), as well as a drug resistance phenotype similar to MDR1 strains (Fig. 6C, D). The *atrB* and *mrr1* mutants showed only little changes in sensitivity to fenhexamid, a substrate for MfsM2 but not AtrB, which confirmed that the functions of AtrB and Mrr1 are fungicide specific. These data confirmed that Mrr1 is the main transcriptional activator of *atrB*, and that activating mutations of *mrr1* lead to overexpression of *atrB* and thus to MDR1 phenotypes. There are interesting parallels to the yeasts *S. cerevisiae* and *C. albicans*, in which MDR-related efflux transporter genes are regulated by Zn(II)_2_Cys_6_ TFs as well. *C. albicans* MDR strains selected by fluconazole treatments in humans also resulted from gain-of-function TF mutations, leading to overexpression of ABC- and MFS-type MDR transporters [13],[30].

### A unique promoter rearrangement leads to MDR2

**Figure 7:**
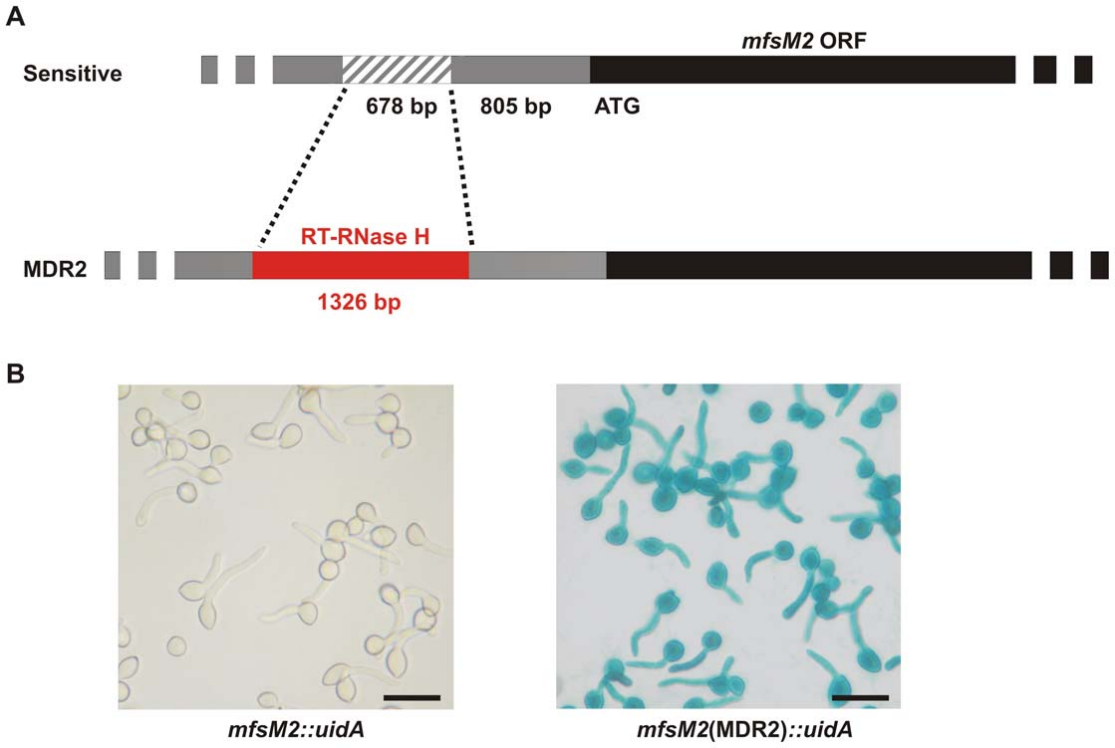
MDR2 strains carry a retroelement-like gene fragment in the *mfsM2* promoter. (A) Structure of the *mfsM2* upstream region, and the insertion-deletion rearrangement (in red) in MDR2 and MDR3 strains. The retroelement-like gene fragment encodes truncated reverse transcriptase (RT) and RNase H domains. The deleted region is indicated as hatched bar. (B) The rearrangement leads to activation of the *mfsM2* promoter. *B. cinerea* transformants carrying *uidA* fusions with *mfsM2* upstream fragments from strain B05.10 and MDR2 strain D08.2-12 were stained for ß-glucuronidase activity. Scale bars: 20 µm.

Focusing on the search for mutations that are responsible for *mfsM2* upregulation in MDR2 and MDR3 strains, we found a rearrangement in the *mfsM2* upstream region, caused by insertion of a foreign gene fragment and concurrent deletion of a portion of the putative *mfsM2* promoter. The inserted DNA, 1326 bp in length, is probably derived from an as yet unknown fungal long-terminal-repeat (*LTR*) retrotransposon (Fig. 7A) [31]. Surprisingly, this sequence is not present in the published genome sequences of the two *B. cinerea* strains B05.10 and T4. It encodes truncated portions of a putative enzyme with domains of reverse transcriptase and RNase H, with closest homologs to sequences in the REAL retrotransposon of the plant pathogenic fungus *Alternaria alternata* [32] and in the Boty retroelement of *B. cinerea* (Fig. S1) [33]. Out of 17 MDR2 and MDR3 strains analyzed from the Champagne (9 strains) and from the German Wine Road (8 strains) by sequencing or PCR analysis, all revealed the identical *mfsM2* promoter rearrangement. In contrast, in 8 sensitive and 15 MDR1 strains no such rearrangement was found (Table S5; data not shown). This observation strongly suggests that the *mfsM2* alleles of these MDR2 and MDR3 strains have a common progenitor. That the promoter rearrangement is responsible for *mfsM2* overexpression, was supported by the already described MDR2-like phenotype of a sensitive strain transformed with an *mfsM2* overexpression construct. This was further confirmed by creating *B. cinerea* strains expressing *mfsM2*::*uidA* reporter gene fusion constructs. Only with the *mfsM2* promoter fragment from an MDR2 strain, but not with a fragment from a sensitive strain, strong expression of ß-glucuronidase was observed (Fig. 7B).

### MDR3 strains are selected by fungicide treatments in the field

**Figure 8:**
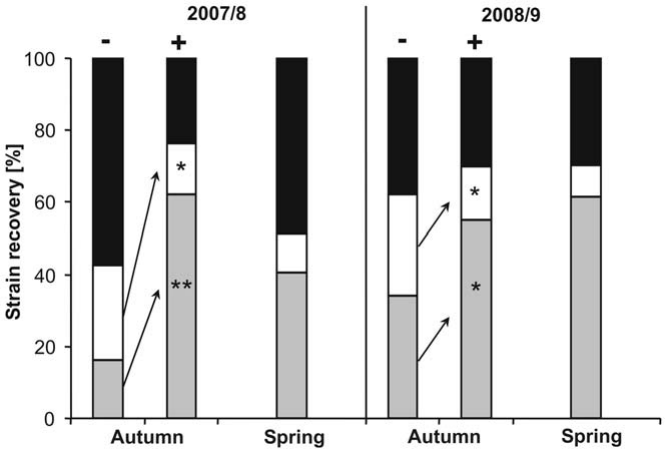
Field competitiveness of an MDR3 strain is increased by fungicide selection. Recovery rates of *B. cinerea* isolates from inoculated grapevine plants during grape harvest (autumn), and in the following spring, in two successive years. Fungicide treated (+) and non-treated (−) grapevine plants were inoculated with a 1∶1 mixture of an MDR3 and a sensitive strain. Grey: Introduced MDR3 strain; White: Introduced sensitive strain; Black: Resident strains. Significantly different recovery rates for the introduced strains are shown.

The rapidly increasing MDR populations in French and German wine-growing regions indicate that strong selection for MDR phenotypes occurs by fungicide treatments. This was confirmed by two field experiments, in which mixtures of an MDR3 strain and a sensitive strain were introduced into two vineyards. A single treatment with a commercial fungicide mixture (fludioxonil and cyprodinil) during early berry development led to a significantly increased recovery of the MDR3 strain relative to the sensitive strain during grape harvest (Fig. 8). The recovery rates of the introduced MDR3 strain were 16% (untreated vineyard) and 62% (treated vineyard) in 2007, and 34% (untreated) and 55% (treated) in 2008, while the recovery rates of the introduced sensitive strain were 26% (untreated) and 14% (treated) in 2007, and 28% (untreated) and 15% (treated) in 2008. In addition, the MDR3 strain showed high survival rates after the following winter periods in the absence of fungicide treatments (40% in spring 2008, 62% in spring 2009). These data indicate also that the mutations in *mrr1* and *mfsM2* do not impair the fitness of MDR strains to a major extent.

## Discussion

Since the introduction of modern fungicides with specific modes of action, resistance development in fungal field populations has been observed, notably in *B. cinerea* which is considered to be a high risk pathogen [2]. Therefore, rules for fungicide resistance management have been established that include a recommendation to avoid the repetitive use of fungicides with similar targets within one growing season [2],[5]. For *Botrytis* control in commercial European vineyards, two or three treatments with different mode-of-action fungicides are common. The increasing and widespread prevalence of MDR strains indicates that they have been selected under these conditions within the last decade.

The three MDR phenotypes found in French and German vineyards were clearly correlated with increased drug efflux activity. Increased fludioxonil efflux was observed for MDR1 and MDR3 strains but not for MDR2 strains, while bitertanol efflux was observed for all MDR phenotypes, although more weakly for MDR1. Furthermore, all MDR strains showed strong constitutive overexpression of one (MDR1, MDR2) or two (MDR3) drug efflux transporter genes. In addition, weak overexpression of two other ABC transporter genes was observed in MDR1 and MDR3 strains. A causal correlation of MDR1 phenotypes with overexpression of the ABC transporter *atrB*, and of MDR2 phenotypes with MFS transporter *mfsM2* overexpression was confirmed by the analysis of deletion and overexpression mutants. Two MDR1 strains with an *atrB* mutation had lost the MDR1 phenotype, and two MDR2 strains with an *mfsM2* mutation had lost the MDR2 phenotypes. In addition, a sensitive strain which artificially overexpressed *mfsM2* showed an MDR2-like phenotype. Thus, overexpression of *atrB* and *mfsM2* are likely to be sufficient for the observed MDR1 and MDR2 phenotypes in *B. cinerea* field strains. While the role of *atrB* in the export of multiple natural and synthetic toxicants has been described in detail [14],[28], the function of *mfsM2* in sensitive strains remains unknown. The protein is not highly conserved in other fungi, and even in the genome sequence of the closely related *Sclerotinia sclerotiorum*, no apparent orthologue to *B. cinerea mfsM2* could be identified. In sensitive strains *mfsM2* expression is very low, and up to now we did not find any fungicide or other compounds that induce *mfsM2* (data not shown).

In MDR1 strains, the mutations leading to *atrB* overexpression were found to be located not in *atrB* itself, but in the transcription factor gene *mrr1*. Out of 20 MDR1 and MDR3 strains analyzed, all carried mutations in the coding region of *mrr1*, and several strains with different geographical origin or collected in different years showed identical mutations. Because *mrr1* mutants failed to express *atrB* to significant levels, and because sensitive strains expressing an activated version of Mrr1 (Mrr1^V575M^) showed an MDR1-like phenotype, Mrr1 was confirmed to be a transcriptional activator of *atrB*. The *mrr1* mutants also showed reduced expression of *atrK* and *BMR3*, when compared to MDR1 strains, indicating that Mrr1 also plays a role in activation of other efflux transporter genes. However, since the drug sensitivity phenotypes of MDR1 strains with either *atrB* or *mrr1* mutations were indistinguishable from each other, and since MDR1 *atrB* mutants have completely lost their MDR1 phenotypes, the weak overexpression of *atrK* and *BMR3* does not seem to contribute significantly to the phenotype of MDR1 strains. While our data indicate that the main physiological role of Mrr1 is regulation of *atrB*, the whole set of genes controlled by Mrr1 in the genome of *B. cinerea* remains to be determined. Interestingly, while structurally and also functionally conserved orthologues of *B. cinerea* AtrB occur in other ascomycetous fungi, e.g. in *A. nidulans*, *P. digitatum* [15],[22], no clear orthologues (best bidirectional hits) of *B. cinerea* Mrr1 could be identified in other fungi. This indicates that the regulation of ABC transporters in filamentous fungi might be not highly conserved. Similar observations have been made for yeasts, because the major efflux transporter in *S. cerevisiae*, PDR5, and its orthologue pair CDR1/CDR2 in *C. albicans*, are under control of different transcription factors [34].

With regard to fungicide resistance, efflux activities, efflux transporter gene overexpression, and genetic data, MDR3 strains were clearly identified as recombinants carrying both MDR1-specific mutations in *mrr1* and MDR2-specific mutations in *mfsM2*. Collectively, all data indicate that the three MDR phenotypes in *B. cinerea* have originated by mutations in just two genes. Obviously, the different *mrr1* point mutations leading to MDR1 have occurred repeatedly. Thus MDR1 phenotypes could appear (and might have already appeared) in different agricultural environments in which selective conditions for these phenotypes prevail. Similarly, a variety of gain-of-function mutations have been found in transcription factor genes *TAC1* and *MRR1* of clinical MDR isolates of *C. albicans*, leading to overexpression of the efflux transporter genes *MDR1* and *CDR1* or *CDR2*, respectively [13],[30],[35]. In contrast, the rearrangement in the *mfsM2* promoter appeared to be a unique event, found in all MDR2 and MDR3 strains analyzed so far. Based on the time course of appearance of these strains in the Champagne, and their lower frequency compared to MDR1 strains in Germany, we assume that the rearrangement in *mfsM2* originated once in the Champagne, possibly in the early 1990s. The rearranged *mfsM2* allele later spread into the German Wine Road region, 250 km east of the Champagne, possibly by air currents. Because of the small size of the retrotransposon-derived gene fragment and of the lack of any remaining LTR sequences, the origin of the sequence inserted into *mfsM2* and the mechanism of the insertion-deletion rearrangement remain obscure. The absence of the integrated sequence in the published genomes of strains B05.10 and T4 indicates that it occurs only in subpopulations of *B. cinerea*. A search for the presence of the sequence in a variety of field strains by using PCR, Southern hybridization and sequencing revealed that similar but non-identical sequences are present in some sensitive strains (data not shown). The *mfsM2* mutation is reminiscent of a transposable element insertion into the promoter of a gene for a cytochrome P450 monooxygenase involved in insecticide detoxification and resistance in *Drosophila*, leading to overexpression and global spread of the mutated gene [36].

**Figure 9:**
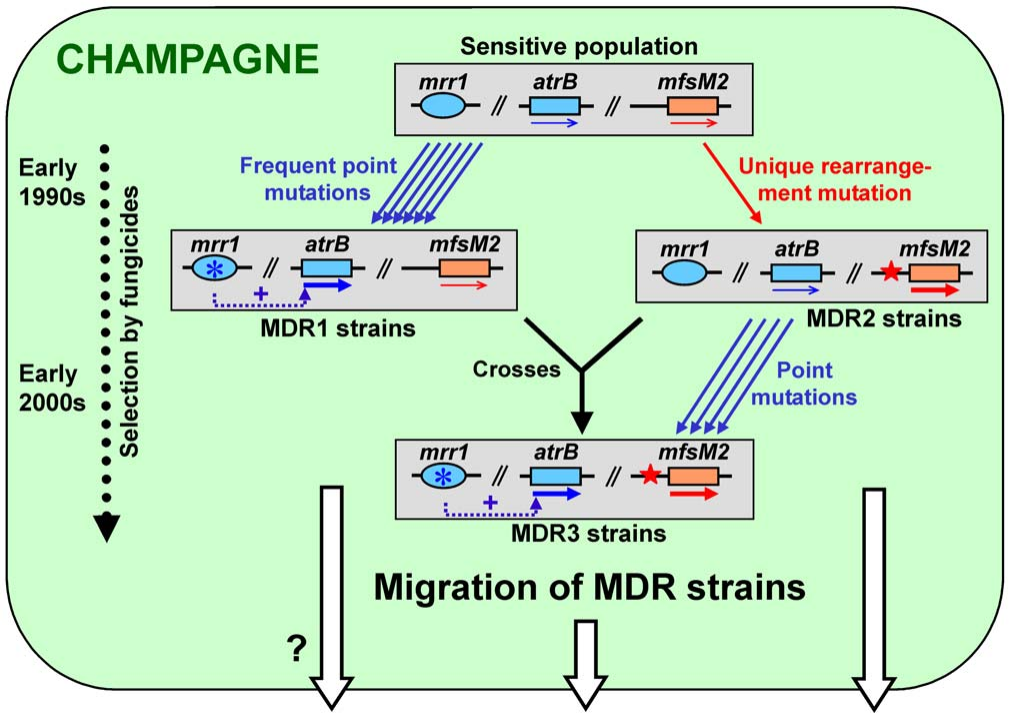
Model for the appearance of MDR phenotypes in *B. cinerea* vineyard populations. Regularly alternating treatments with modern fungicides, in particular the anilinopyrimidines pyrimethanil and cyprodinil (since 1990), the phenylpyrrole fludioxonil (since 1995), and the hydroxyanilide fenhexamid (since 2000) are assumed to be responsible for the selection of MDR phenotypes in Champagne vineyards. Repeatedly occurring point mutations in the transcription factor gene *mrr1* (blue rose symbol) lead to overexpression of the ABC transporter gene *atrB* (unlinked to *mrr1*) and thus to MDR1 phenotype. In contrast, a unique promoter rearrangement in the MFS transporter gene *mfsM2* (red asterisk) is responsible for its overexpression and MDR2 phenotype. Strains with MDR3 phenotype, carrying both types of mutations and showing increased MDR, might have originated either by natural MDR1×MDR2 crosses or by secondary *mrr1* mutations in MDR2 strains. MDR2 and MDR3 strains, and possibly also MDR1 strains, have migrated within and out of the Champagne, reaching at least the German Wine Road region, 250 km east of the Champagne. Further evidences for the migration are the delayed appearance of MDR2/3 strains in Germany, and the failure until now to detect them in France outside of the Champagne. Gene expression is indicated by arrows, bold arrows indicate overexpression, and the dotted arrows with the ‘+’ sign indicate transcription factor-mediated activation.

A summarizing model of the data in this paper is shown in Fig. 9. It is assumed that MDR strains in the Champagne have appeared due to selection pressure in fungicide treated vineyards, and to mutations leading to the appearance of MDR1 and MDR2 strains, and a few years later also to the appearance of MDR3 strains. Because a repeated occurrence of the unusual rearrangement found in the *mfsM2* promoter appears to be highly unlikely, we assume that MDR2 strains carrying this rearrangement have migrated from France to Germany, probably in the last decade. To support this hypothesis, population genetic studies with MDR strains from different geographical origins are currently performed. In addition, we are searching for MDR strains in other regions with different crop cultures and different fungicide treatment schedules, in order to achieve a better understanding of the distribution of MDR strains, and the factors leading to their appearance and selection.

A field experiment has clearly demonstrated selection of an artificially introduced MDR3 strain by a standard fungicide treatment. This confirms that the MDR3 phenotype confers selective advantage to *B. cinerea* in fungicide-treated vineyards, and that this advantage outweighs possible fitness defects. Furthermore, the MDR3 strain was recovered with high albeit varying frequencies after overwintering periods, in the absence of fungicide selection pressure. These data indicate that the general fitness of strains showing *atrB* and/or *mfsM2* overexpression can be rather high in field environments, but this needs further studies. We are currently testing various fitness parameters in isogenic *atrB*, *mrr1* and *mfsM2* knock-out and overexpression strains in order to estimate the performance of these strains. Because the natural role of MDR-related efflux transporters seems to be the protection against various biotic toxic compounds [14],[26],[28], it is possible that the MDR strains have acquired properties that increase their fitness in natural environments even in the absence of fungicides.

Our work is the first documented case of a massive appearance of MDR populations in a major plant pathogen in fungicide-treated agricultural environments. To what extent the effectiveness of fungicide treatments against MDR strains is reduced in comparison to sensitive strains needs to be investigated. Nevertheless, the possibilities of a further rise of MDR3 strains and of additional mutations leading to higher levels of broad-spectrum fungicide resistance are expected to be a major threat for chemical control of grey mould disease in the near future.

## Methods

### Isolation and cultivation of *B. cinerea* strains

Strains were isolated from commercial vineyards in the Champagne and the German Wine Road (Palatinate). In the Champagne, samples were collected from vineyards located around Moulins, Hautvillers, Vandières and Courteron, Moulins being the northernmost (49°34′N/03°28′E) and Courteron the southernmost (48°01′N/04°26′E) town, 167 km apart from each other. Samples were collected from approximately 200 locations each year. Each sample represented a bulk population consisting of spores of at least 20 infected berries within the chosen plot. The spores from each bulk sample were spread onto agar media containing different fungicides, and analyzed for different phenotypes as described [25],[37].

In the German Palatinate, the same six vineyards were used for sampling each year. The plots are located along the German Wine Road, between Dackenheim (northernmost: 49°53′N/8°19′E) and Walsheim (southernmost: 49°23′N/8°13′E), 32 km apart from each other. Thirty isolates were obtained per vineyard from single infected berries, resulting in about 180 isolates per year. From each sample, HA (1% (w/v) malt extract, 0.4% (w/v) yeast extract, 0.4% (w/v) glucose, pH 5.5) cultures were grown, and terminal mycelial fragments cut off for subculture of isolates. Conidia were used for fungicide tests. A list of *B. cinerea* strains is shown in Table S5.

### Fungicide sensitivity tests

Fludioxonil, cyprodinil (Syngenta-Agro, Maintal, Germany), fenhexamid, tebuconazole (Bayer Crop Sciences, Monheim, Germany), boscalid, iprodione (BASF, Ludwigshafen, Germany), were kindly provided by the companies, carbendazim, tolnaftate and cycloheximide were purchased from Sigma-Aldrich (St. Louis, USA). The drugs were dissolved in 100% ethanol or 100% DMSO (carbendazim), and added to the required concentrations to the assays. For dilution series, fungicide stock solutions were adjusted to keep the final solvent concentrations between 0.2 and 1.5% (v/v) for ethanol and between 0.3 and 1.0% (v/v) for DMSO. Control assays revealed no significant differences in growth of the strains at these concentrations relative to no-solvent controls (not shown). For each isolate tested, 2×10^5^ conidia were pre-incubated for 1.5 hours in 1 ml malt extract broth (pH 5.5; Difco) before use. Effective inhibitory drug concentrations (EC_50_; mg/l) were determined with 1000 spores in 0.1 ml 96-microplate cultures, using threefold drug dilution series. Tests were performed in malt extract broth, except for cyprodinil (Gamborg B5 minimal medium supplemented with 10mM KH_2_PO_4_, 50mM glucose; pH 5.5), and boscalid [38]. After 48 h (boscalid: 96 h) incubation at 20°C, A_600_ was determined. The assays were repeated at least 3 times. Mean data, with standard deviations are presented. For calculation of EC_50_ values, the Origin6.0 software package (Origin Lab Cooperation, USA) was used.

### Accumulation of ^14^C-labeled fungicides

Fungicide accumulation assays with ^14^C-labeled fludioxonil and bitertanol were performed with 14 h old germlings germinated as described previously [27]. Experiments were initiated by adding the labeled fungicide to final concentrations of 6 µM (10 Bq/nmol) fludioxonil, or 10 µM (10 Bq/nmol) bitertanol. The uncoupler CCCP was added at a final concentration of 10 µM. Three 5 ml samples each were taken 10 and 60 min after adding the fungicide. Heat inactivation of germlings for control experiments was performed for 10 min at 60°C. Experiments were done in triplicates and repeated at least three times.

### DNA and RNA manipulations and measurements

DNA isolation and manipulation was performed according to established protocols. For transcript studies, *B. cinerea* conidia (2×10^6^) were germinated for 15 h in polystyrene Petri dishes coated with apple wax (0.01 mg/cm^2^) using Gamborg B5 medium supplemented with 10 mM fructose and 10 mM KH_2_PO_4_ (pH 5.5). The germlings were incubated for further 30 min either without or with 1 mg/l fludioxonil. For RNA isolation, the wax with the embedded germlings was scraped from the surfaces with a tissue cell scraper (TPP AG, Trasadingen, Switzerland), centrifuged for 5 min at 4000 rpm at 4°C, washed with 20 ml of ice-cold water and centrifuged once more. The pellet was transferred into a mortar containing liquid nitrogen and sea sand for grinding. Total fungal RNA was isolated using the RNeasy Plant Mini Kit (Qiagen, Hilden, Germany), and reverse transcribed into cDNA with oligo(dT) primers (Verso cDNA Kit; Thermo Fisher Scientific, Surrey, United Kingdom). Northern hybridization and quantitative RT-PCR were performed according to standard protocols. Expression of the genes was calculated according to Pfaffel [39]. Transcript levels were normalised against the expression levels of housekeeping genes encoding elongation factor 1α (BC1G_09492.1) and actin (BC1G_08198.1), and shown as normalized fold-expression relative to expression levels of non-induced germlings from sensitive strains. Means of at least two biological replicates, with three strains of each phenotype, are shown.

The following efflux transporter genes were analyzed: (*Bc*)*atrB* [16], (*Bc*)*atrD* [18], (*Bc*)*atrA* [40], (*Bc*)*atrF* (BC1G_01454.1), (*Bc*)*atrK*/*BMR1* [16], *BMR3* (BAC67160; BC1G_02799) [41], (*Bc*)*mfsM2* (BofuT4_P024110.1). For identification of mutations in the *mrr1* alleles of sensitive, MDR1 and MDR3 strains, *mrr1* fragments were amplified from total DNA by PCR, using primers mrr1_TF1-1 and mrr1_TF1-4, and sequenced. For identification of the *mfsM2* alleles in sensitive and MDR2/MDR3 strains, the *mfsM2* upstream region was amplified from genomic DNA by primers mfsM2-pfor/mfsM2-prev, yielding a 1625 bp fragment with sensitive strains, and a 2273 bp fragment with MDR2 and MDR3 strains. For confirmation of their identity, the insertions of 6 MDR2 strains were sequenced.

### Generation of *B. cinerea* knock-out, overexpression and reporter fusion mutants

For *atrB* mutagenesis, the construct described by Vermeulen et al. [16] was used. For *mrr1* deletion, a genomic *B. cinerea mrr1* fragment was amplified (primers mrr1-for1/mrr1-rev2), digested (*Apa*I/*Sac*II) and cloned into pBSKS(+). Inverse PCR was performed (primers mrr1-rev1/mrr1-for2), the product digested (*Eco*RV/*Xma*I) and ligated with a hygromycin cassette [42]. After transformation into *B. cinerea* [43], mutants were identified with primers mrr1-for1/tubB-inv (yielding a 1304 bp product in k.o. mutants). For *mfsM2* deletion, two *mfsM2* flanking fragments were amplified (1: mfsM2-KO1/mfsM2-KO2; 2: mfsM2-KO3/mfsM2-KO4), digested (1: *Xba*I/*Eco*RI; 2: *Kpn*I/*Xho*I), and successively cloned into pBSKS(+). The hygromycin cassette from pLOB1 (AJ439603) was inserted between the fragments via *Eco*RI and *Xho*I. The construct was amplified (primers mfsM2KO/mfsM2-KO4) and transformed into MDR2 strains D06.6-5 and D06.2-6. The *ΔmfsM2* mutants were confirmed by PCR (primers mfsM2-KO1/oliC-Sma-Rev), yielding a 1605 bp product in k.o. mutants. To construct strains expressing an *mrr1* allele conferring MDR1, *mrr1^V575M^* of strain D06.7-27 was amplified (primers mrr1-atg/mrr1-uaa), digested (*Xma*I/*Pvu*II) and cloned into pBSKS(+) carrying a 5′-fragment of the hygromycin resistance gene driven by the *oliC* promoter [44]. Additional 1578 bp of the *mrr1* upstream region were amplified (primers mrr1-pro1/mrr1-rev1), digested (*Bam*HI/*Xma*I) and ligated next to *mrr1^V575M^*. The resulting plasmid was linearized (*Kpn*I) and transformed into *B. cinerea* B05.Hyg-3 [44]. To generate *mfsM2* overexpression strains (*mfsM2^ox^*), *mfsM2* was fused to the *oliC* promoter. The *mfsM2* coding sequence was amplified (primers mfsM2-ATG-SmaI/mfsM2-TAG-EcoRI), digested (*Sma*I/*Eco*RI) and ligated into poliGUS-Hyg5, replacing *uidA*. poliGUS-Hyg5 was constructed by fusing an *oliC* promoter fragment from pLOB1 (primers KO-Hyg1-BamHI/oliC-Sma-Rev), an *uidA* coding sequence from p35S-GUS [45] (primers 35S-gus-for-Sma/35S-gus-rev-Eco), the *B. cinerea niaD* terminator (primers niaDTerm-for-Eco/niaDTerm-rev-Hind) and the 5′ part of a splitted hygromycin resistance cassette from pBS.Hyg-5 [44] (*Hin*dIII/*Xho*I) into pBSKS(+). The resulting plasmid was linearized (*Kpn*I) and transformed into strain B05.Hyg-3, yielding B05.Hyg-3(*mfsM2^ox^*). To construct *mfsM2* promoter-reporter fusions, the *oliC* promoter fragment in poliGUS-Hyg5 was replaced either by a 1501 bp *mfsM2* upstream fragment from strain B05.10, or by a 2149 bp fragment from MDR2 strain D08.2-12 including the 1326 bp retrotransposon-derived fragment and the remaining *mfsM2* upstream region (primers mfsM2-pfor-Not/mfsM2-prev-Sma), before transformation into B05.Hyg-3. *B. cinerea* B05.Hyg-3 transformants were analyzed for correct genomic integration of the constructs by PCR. GUS staining of transformants was performed as described [46].

### Field competitiveness of an MDR strain in a fungicide-treated vineyard

MDR3 strain D06.7-33 and the sensitive (Ben^R^) strain D06.5-25 were chosen for a mixed-inoculation experiment, performed twice in 2007 and 2008 in experimental vineyards at the German Wine Road (Neustadt an der Weinstrasse). In June (berry stage BBCH-77) the vineyards received a standard treatment with Teldor. In summer, immature grapes (stage BBCH-81) were inoculated, using hand spraying bottles, with a 1∶1 strain mixture (2×10^4^ conidia/ml per strain) in water until runoff. The vineyards were randomly divided into three fungicide-treated and three untreated plots. The first inoculation was one day before, the second one day after standard treatment with Switch in the treated plots, and in the same way in non-treated plots. Before grape harvest at late September, 50 (2007) and 90 (2008) isolates per plot were recovered from moulded berries from the inoculated plots and from a non-inoculated, untreated control plot nearby. The introduced isolates were identified by fungicide tests, using HA plates containing 0.2 mg/l fludioxonil, 5 mg/l iprodione, or 5 mg/l carbendazim. In the control plot, MDR3 strains were never detected, while Ben^R^ strains were found with frequencies of 12% (2007) and 6% (2008). Their genetic identity was further confirmed by IGS-AFLP markers [31]. For analysis of the overwintered populations, *B. cinerea* isolates were recovered in the following spring from bark fragments of inoculated grapevines (2008: 137; 2009: 313 isolates), by incubation on a selection medium for *B. cinerea* [47], followed by fungicide tests.

### Statistics

Experiments were performed at least three times, unless indicated otherwise. Statistical differences of data were checked by unpaired, two-tailed t tests, and labeled as follows: n.s.: not significant; * p<0.05; ** p<0.01; *** p<0.001. In the graphs, standard deviations are indicated.

### Accession numbers

The DNA sequence reported in this paper has been deposited in GenBank, under accession number GQ292709 (RE-like gene fragment inserted in *mfsM2*). Further accession numbers: *mfsM2*: BofuT4_P024110.1; *mrr1*: BofuT4_P063510.1 (http://urgi.versailles.inra.fr/projects/Botrytis/).

## Supporting Information

Figure S1 Similarity between the retroelement-like sequences in the *mfsM2* promoter of *B. cinerea* MDR2 and MDR3 strains and in other fungal retrotransposons. Alignment of the translated retroelement-derived gene fragment in the mfsM2 promoter region of MDR2 and MDR3 strains with predicted reverse transcriptase-RNase H sequences from the REAL [32] (*Alternaria alternata*; acc. BAA24352; 551 amino acids) and Boty [33] (*B. cinerea*; acc. XP_001548698; 1618 amino acids) retrotransposons. Conserved reverse transcriptase (RT) and RNase H domains are marked. Dashes: No amino acids.
(0.54 MB TIF)

Table S1 *B. cinerea* crosses. Crosses were performed for map-based cloning of the MDR1 regulator, mrr1 (multidrug resistance regulator 1), and mapping of *mfsM2* (major facilitator superfamily transporter involved in MDR2) in MDR2 and MDR3 strains. Conformity of the observed segregation data with the involvement of single dominant genes for MDR1 (cross 1) and MDR2 (cross 2), and for two independently segregating, codominant genes for MDR1, MDR2 and MDR3 (crosses 3, 4) was analyzed by χ^2^ test, with χ^2^ values calculated for p = 0.05. #Consistent with segregation of two co-dominant genes. Cross 4 showed a deviation from the expected 1∶1∶1∶1 segregation. The reason for this is not clear. An explanation could be unequal survival of the progeny strains, because some of the strains analyzed showed very slow growth.
(0.04 MB RTF)

Table S2 Molecular markers showing linkage of *mrr1* with MDR1 phenotype. The markers were generated by searching for polymorphic microsatellites (MS) in the genomes of *B. cinerea* strains B05.10 (http://www.broad.mit.edu/) and T4 (http://urgi.versailles.inra.fr/gbrowse/cgi-bin/gbrowse/BOTRYTIS_T4). Length polymorphisms of the PCR-amplified marker fragments between the parent strains of crosses 1, 3 and 4 were detected by agarose gel electrophoresis. A total of 24 polymorphic MS markers were initially used for screening the F1 progeny strains derived from crosses 1, 3 and 4, revealing markers BC218 and BC274 as cosegregating with MDR1. Markers BC294-2 and BC63-17 were subsequently generated for fine-mapping of the MDR1 locus. Because mutations leading to overexpression of efflux transporter genes and MDR in *Candida albicans* have been located in transcription factor genes [13],[30], the transcription factor gene *mrr1* which showed the closest linkage with MDR1 in the *B. cinerea* genome, was selected for further analysis. *Fragments obtained after digestion with *Sac*I.
(0.03 MB RTF)

Table S3 Molecular markers confirming linkage of *mfsM2* to MDR2 and MDR3 phenotypes. Based on evidence that *mfsM2* mutations are responsible for the appearance of MDR2 and MDR3 phenotypes, markers located close to *mfsM2* were analyzed. The markers are polymorphic between the parent strains of the indicated crosses. * Fragments obtained after digestion with *Hind*III.
(0.03 MB RTF)

Table S4 Sequence polymorphisms of *mrr1* in *B. cinerea* strains with different MDR phenotypes. All nucleotide exchanges (top row) in the *mrr1* coding region leading to amino acid changes (second row) and silent exchanges relative to the sequences of the sensitive reference strains B05.10 and T4 are shown. To the strain names, the phenotypes are added. Δ23A24P: 6 bp deletion in the *mrr1* coding region, leading to deletion of two codons encoding Ala and Pro. Seven sensitive field strains from Palatinate vineyards had the same Mrr1 sequence as strains T4 and B05.10. Neither the Δ23A24P deletion in several but not all MDR1 strains, nor the conservative V227I exchange in MDR2 strain IXa14 are likely to alter the properties of Mrr1.
(0.20 MB RTF)

Table S5 *B. cinerea* strains used in this study. Ben^R^, Imi^R^: Strains resistant to benzimidazoles and the dicarboximide iprodione, respectively. If tested, the *mrr1* and the *mfsM2* alleles are indicated. Hyg^R^, Phleo^R^: Transformation-mediated resistance to hygromycin and phleomycin, respectively. ^1^Derived from a cross between strains 4.33.10b (MDR1, isolated in the Champagne in 1994) and SAS56. ^2^Derived from a cross between strains SAS405 and B.692 (MDR2, isolated in the Champagne in 1994) and strain SAS56. ^3^Site of isolation within the Champagne unknown. n.t.: not tested. Rearrangement of the *mfsM2* promoter was tested either by sequencing (*mfsM2* ^seq(+)^: rearranged; *mfsM2* ^seq(−)^: not rearranged) or by PCR (*mfsM2* ^pcr(+)^ or *mfsM2* ^pcr(−)^).
(0.15 MB RTF)

Table S6 Oligonucleotide primers used in this study. Introduced restriction sites are underlined.
(0.08 MB RTF)
