# Supplementary figures and images for: Fungicide-Driven Evolution and Molecular Basis of Multidrug Resistance in Field Populations of the Grey Mould Fungus Botrytis cinerea
Source: PLoS Pathog. 2009 Dec 18;5(12):e1000696. doi: 10.1371/journal.ppat.1000696 (PMC2785876; doi:10.1371/journal.ppat.1000696)

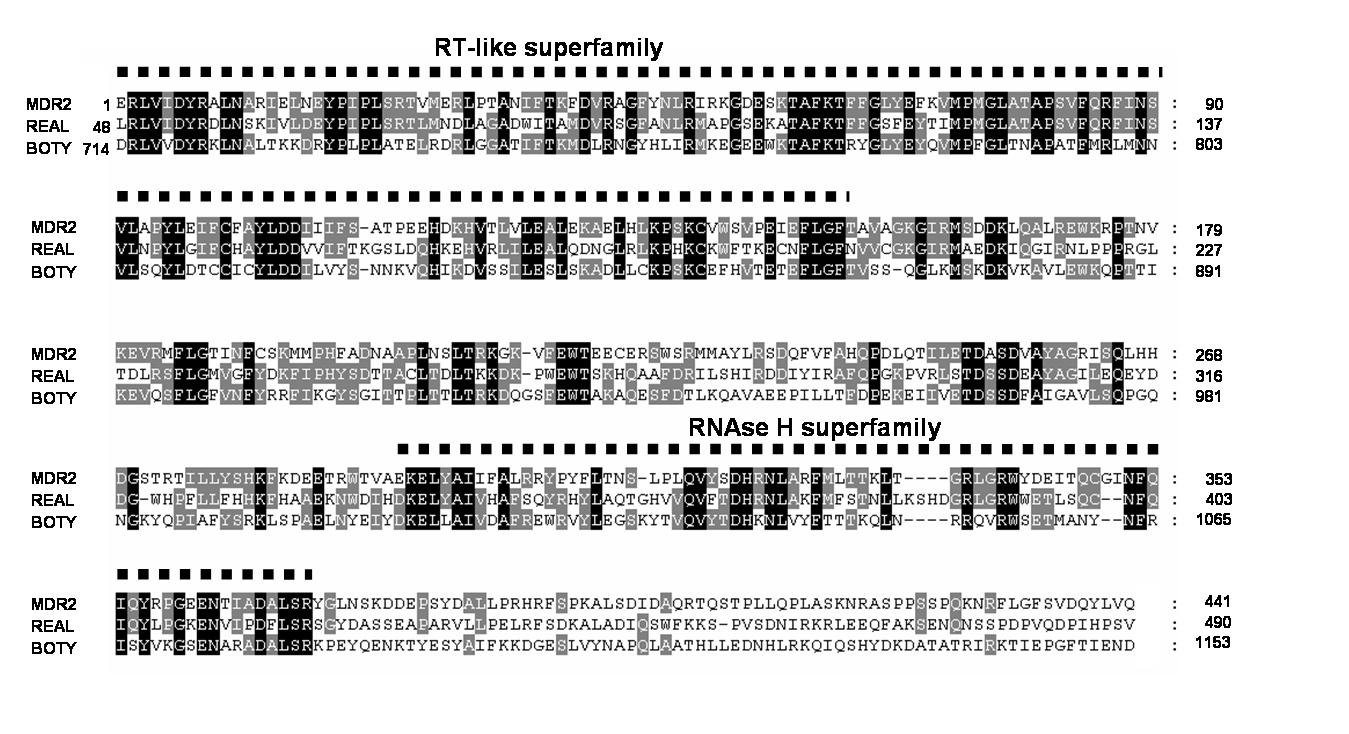

Supplement: Figure S1 — Similarity between the retroelement-like sequences in the mfsM2 promoter of B. cinerea MDR2 and MDR3 strains and in other fungal retrotransposons. Alignment of the translated retroelement-derived gene fragment in the mfsM2 promoter region of MDR2 and MDR3 strains with predicted reverse transcriptase-RNase H sequences from the REAL [32] (Alternaria alternata; acc. BAA24352; 551 amino acids) and Boty [33] (B. cinerea; acc. XP_001548698; 1618 amino acids) retrotransposons. Conserved reverse transcriptase (RT) and RNase H domains are marked. Dashes: No amino acids. (0.54 MB TIF) [file ppat.1000696.s001.tif]
