# Supplementary material for: Fungicide-Driven Evolution and Molecular Basis of Multidrug Resistance in Field Populations of the Grey Mould Fungus Botrytis cinerea
Source: PLoS Pathog. 2009 Dec 18;5(12):e1000696. doi: 10.1371/journal.ppat.1000696 (PMC2785876; doi:10.1371/journal.ppat.1000696)
Supplement: Table S1 — B. cinerea crosses. Crosses were performed for map-based cloning of the MDR1 regulator, mrr1 (multidrug resistance regulator 1), and mapping of mfsM2 (major facilitator superfamily transporter involved in MDR2) in MDR2 and MDR3 strains. Conformity of the observed segregation data with the involvement of single dominant genes for MDR1 (cross 1) and MDR2 (cross 2), and for two independently segregating, codominant genes for MDR1, MDR2 and MDR3 (crosses 3, 4) was analyzed by χ2 test, with χ2 values calculated for p = 0.05. #Consistent with segregation of two co-dominant genes. Cross 4 showed a deviation from the expected 1∶1∶1∶1 segregation. The reason for this is not clear. An explanation could be unequal survival of the progeny strains, because some of the strains analyzed showed very slow growth. (0.04 MB RTF) [file ppat.1000696.s002.rtf]

Cross	Parent 1
(spermatial)	Parent 2
(sclerotial)	No. of F1 isolates	Phenotypes of F1 isolates (%)	Calculated (expected)
÷2 value	 Reference	
				 Sens.	 MDR1	 MDR2	 MDR3			
1	6.220a 
(MDR1)	SAS56 (sensitive)	74	50.0	50.0	-	-	0 (3.84)#	25	
2	SAS405 (sensitive)	6146c 
(MDR2)	97	45.4	-	54.6	-	0.84 (3.84)#	25	
3	IVa2 
(MDR1)	IXa14 
(MDR2)	92	23.9	30.4	19.6	26.1	2.26 (7.81)#	This work	
4	F02.392 
(MDR3)	SAS56 (sensitive)	73	21.9	21.9	16.4	39.7	9.03 (7.81)	This work	
