# Supplementary material for: Fungicide-Driven Evolution and Molecular Basis of Multidrug Resistance in Field Populations of the Grey Mould Fungus Botrytis cinerea
Source: PLoS Pathog. 2009 Dec 18;5(12):e1000696. doi: 10.1371/journal.ppat.1000696 (PMC2785876; doi:10.1371/journal.ppat.1000696)
Supplement: Table S2 — Molecular markers showing linkage of mrr1 with MDR1 phenotype. The markers were generated by searching for polymorphic microsatellites (MS) in the genomes of B. cinerea strains B05.10 (http://www.broad.mit.edu/) and T4 (http://urgi.versailles.inra.fr/gbrowse/cgi-bin/gbrowse/BOTRYTIS_T4). Length polymorphisms of the PCR-amplified marker fragments between the parent strains of crosses 1, 3 and 4 were detected by agarose gel electrophoresis. A total of 24 polymorphic MS markers were initially used for screening the F1 progeny strains derived from crosses 1, 3 and 4, revealing markers BC218 and BC274 as cosegregating with MDR1. Markers BC294-2 and BC63-17 were subsequently generated for fine-mapping of the MDR1 locus. Because mutations leading to overexpression of efflux transporter genes and MDR in Candida albicans have been located in transcription factor genes [13],[30], the transcription factor gene mrr1 which showed the closest linkage with MDR1 in the B. cinerea genome, was selected for further analysis. *Fragments obtained after digestion with SacI. (0.03 MB RTF) [file ppat.1000696.s003.rtf]

Marker (distance 
from mrr1)	Crosses used
(no. of progeny)	Approx. PCR fragment sizes (bp) in parent strains	Linkage to MDR1 (%)	
		6.220a	SAS56	IVa2	IXa14	F02.392A		
BC218 (746 kb)	1, 3, 4 (232)	240	230	230	210	245	66.0	
BC274 (134 kb)	1, 3, 4 (232)	210	290	210	280	270	90.4	
BC294-2 (22.5 kb)	1, 3, 4 (232)	1300*	900+400*	1000+300*	900+400*	1300*	94.6	
BC63-17 (1.8 kb)	1, 3, 4 (232)	750	780	240	250	250	100	
