# Supplementary material for: Fungicide-Driven Evolution and Molecular Basis of Multidrug Resistance in Field Populations of the Grey Mould Fungus Botrytis cinerea
Source: PLoS Pathog. 2009 Dec 18;5(12):e1000696. doi: 10.1371/journal.ppat.1000696 (PMC2785876; doi:10.1371/journal.ppat.1000696)
Supplement: Table S3 — Molecular markers confirming linkage of mfsM2 to MDR2 and MDR3 phenotypes. Based on evidence that mfsM2 mutations are responsible for the appearance of MDR2 and MDR3 phenotypes, markers located close to mfsM2 were analyzed. The markers are polymorphic between the parent strains of the indicated crosses. * Fragments obtained after digestion with HindIII. (0.03 MB RTF) [file ppat.1000696.s004.rtf]

Marker (distance from mfsM2)	Cross used
(no. of progeny)	Approx. PCR fragment sizes (bp) in parents	Linkage to 
MDR2 (%) 	
		SAS405	6.146c	IVa2	IXa14		
BC302 (192 kb)	3 (92)	-	-	480	470	84.8	
BC13-116 (25.5 kb)	2 (97)	750*	350+400*	-	-	95.9	
BC13-91 (1.2 kb)	3 (92)	-	-	380	330	100	
