# Supplementary material for: Fungicide-Driven Evolution and Molecular Basis of Multidrug Resistance in Field Populations of the Grey Mould Fungus Botrytis cinerea
Source: PLoS Pathog. 2009 Dec 18;5(12):e1000696. doi: 10.1371/journal.ppat.1000696 (PMC2785876; doi:10.1371/journal.ppat.1000696)
Supplement: Table S4 — Sequence polymorphisms of mrr1 in B. cinerea strains with different MDR phenotypes. All nucleotide exchanges (top row) in the mrr1 coding region leading to amino acid changes (second row) and silent exchanges relative to the sequences of the sensitive reference strains B05.10 and T4 are shown. To the strain names, the phenotypes are added. Δ23A24P: 6 bp deletion in the mrr1 coding region, leading to deletion of two codons encoding Ala and Pro. Seven sensitive field strains from Palatinate vineyards had the same Mrr1 sequence as strains T4 and B05.10. Neither the Δ23A24P deletion in several but not all MDR1 strains, nor the conservative V227I exchange in MDR2 strain IXa14 are likely to alter the properties of Mrr1. (0.20 MB RTF) [file ppat.1000696.s005.rtf]

	Ä65-70	G819A	T752C	T993C	C994T	G1155C	G1220A	A1590G	G1697A	G1723A	G1832A	G1858A	G1895T	G2025T	A2033C	C2118T	C2190T	
	Ä23A24P	V227I	M251T	silent	L332F	D385H	R407Q	silent	G566Q	V575M	S611 R	G620R	S632 R	M675I	N678T	silent	silent	
(Sensit. strains)	-	-	-	-	-	-	-	-	-	-	-	-	-	-	-	-	C/T	
IXa14_MDR2	-	+	-	-	-	-	-	-	-	-	-	-	-	-	-	-	C	
6220a_MDR1	+	-	-	-	-	-	+	-	-	-	-	-	-	-	-	-	T	
IVa2_MDR1	-	-	-	-	-	-	-	-	-	+	-	-	-	-	-	-	c	
D04.375_MDR1	+	-	-	-	-	-	-	+	-	-	+	-	-	-	-	-	t	
D06.2-2_MDR1	-	-	-	-	-	-	-	-	-	+	-	-	-	-	-	-	c	
D06.3-4_MDR1	+	-	-	-	-	-	-	-	-	-	-	+	-	-	-	+	t	
D06.3-27_MDR1	+	-	-	-	-	-	-	-	-	-	+	-	-	-	-	-	t	
D06.5-16_MDR1	+	-	-	-	-	-	-	-	-	-	-	-	+	-	-	-	t	
D06.6-17_MDR1	-	-	+	+	+	-	-	-	-	-	-	-	-	-	-	-	c	
D06.6-22_MDR1	+	-	-	-	-	-	-	-	-	-	-	+	-	-	-	-	t	
D06.7-27_MDR1	+	-	-	-	-	-	-	-	-	+	-	-	-	-	-	-	t	
D08.2-10_MDR1	-	-	+	-	-	-	-	-	-	-	-	-	-	-	-	-	c	
D08.3-17_MDR1	+	-	-	-	-	-	+	-	-	-	-	-	-	-	-	-	t	
D08.4-28_MDR1	+	-	-	-	-	-	-	-	-	-	+	-	-	-	-	-	t	
D08.5-12_MDR1	-	-	-	-	-	-	-	-	-	-	-	+	-	+	+	-	c	
D08.6-15_MDR1	-	-	-	-	-	-	-	-	+	-	-	-	-	-	-	-	t	
F96.F31_MDR3	-	-	-	-	-	+	-	-	-	-	-	-	-	-	-	-	c	
F96.F33_MDR3	-	-	-	-	-	-	-	-	-	-	-	+	-	-	-	-	c	
D06.7-33_MDR3	-	-	-	-	-	-	-	-	-	-	-	+	-	-	-	-	c	
D08.2-2_MDR3	-	-	-	-	-	-	-	-	-	-	-	+	-	-	-	-	c	
D08.6-26_MDR3	-	-	-	-	-	-	-	-	-	+	-	-	-	-	-	-	c	
