# Supplementary material for: Fungicide-Driven Evolution and Molecular Basis of Multidrug Resistance in Field Populations of the Grey Mould Fungus Botrytis cinerea
Source: PLoS Pathog. 2009 Dec 18;5(12):e1000696. doi: 10.1371/journal.ppat.1000696 (PMC2785876; doi:10.1371/journal.ppat.1000696)
Supplement: Table S5 — B. cinerea strains used in this study. BenR, ImiR: Strains resistant to benzimidazoles and the dicarboximide iprodione, respectively. If tested, the mrr1 and the mfsM2 alleles are indicated. HygR, PhleoR: Transformation-mediated resistance to hygromycin and phleomycin, respectively. 1Derived from a cross between strains 4.33.10b (MDR1, isolated in the Champagne in 1994) and SAS56. 2Derived from a cross between strains SAS405 and B.692 (MDR2, isolated in the Champagne in 1994) and strain SAS56. 3Site of isolation within the Champagne unknown. n.t.: not tested. Rearrangement of the mfsM2 promoter was tested either by sequencing (mfsM2 seq(+): rearranged; mfsM2 seq(−): not rearranged) or by PCR (mfsM2 pcr(+) or mfsM2 pcr(−)). (0.15 MB RTF) [file ppat.1000696.s006.rtf]

Strain	Origin	Phenotypes / Genotypes	Reference	
B05.10	laboratory strain	Sensitive, BenR	48	
B05.10(ÄatrB)	Laboratory strain	Sensitive, BenR,  HygR	49	
B05.10(Ämrr1)	Laboratory strain	Sensitive, BenR,  HygR	This work	
B05.Hyg-3	laboratory strain	Sensitive, BenR, PhleoR	44	
B05.Hyg-3 (mrr1V575M)	Laboratory strain	MDR1-like, BenR, PhleoR, HygR	This work	
B05.Hyg-3 (mfsM2ox)	Laboratory strain	MDR2-like, BenR, PhleoR, HygR	This work	
B05.Hyg-3 (mfsM2:uidA)	Laboratory strain	Sensitive, BenR, PhleoR, HygR	This work	
B05.Hyg-3 (mfsM2(MDR2)::uidA)	Laboratory strain	Sensitive, BenR, PhleoR, HygR	This work	
SAS56	Italy	Sensitive	50	
SAS405	Italy	Sensitive, BenR, ImiR	50	
IVa21	F-Champagne3 (1994)	MDR1, Mrr1V575M, mfsM2seq(-), BenR	25	
IXa142	F-Champagne3 (1994)	MDR2, mfsM2seq(+), BenR	25	
6.220a	F-Champagne3 (1994)	MDR1, Mrr1R407Q, mfsM2seq(-), BenR	25	
6.146c	F-Champagne3 (1994)	MDR2, mfsM2pcr(+), BenR	25	
D04.375	D-Freiburg (2004)	MDR1, Mrr1S611R, mfsM2seq(-)	51	
D04.104	D-Freiburg (2004)	MDR1, Mrr1 n.t., BenR	51	
F96.F31	F-Champagne3 (1996)	MDR3, Mrr1D385H, mfsM2pcr(+), BenR	This work	
F96.F33	F-Champagne3 (1996)	MDR3, Mrr1G620R, mfsM2pcr(+), BenR	This work	
F02.392	F-Moulins (2002)	MDR3, Mrr1 n.t., mfsM2pcr(+), BenR, ImiR	This work	
F05.A632 	F-Hautvillers (2005)	MDR3, Mrr1 n.t., mfsM2pcr(+), BenR	This work	
D06.2-2	D-Ruppertsberg (2006)	MDR1, Mrr1V575M, mfsM2pcr(-)	This work	
D06.2-6	D-Ruppertsberg (2006)	MDR2, mfsM2seq(+), BenR 	This work	
D06.2-6(ÄmfsM2)	Laboratory strain	Sensitive, BenR, HygR	This work	
D06.3-4	D-Wachenheim (2006)	MDR1, Mrr1G620R, mfsM2pcr(-)	This work	
D06.3-27	D-Wachenheim (2006)	MDR1, Mrr1S611R, mfsM2pcr(-)	This work	
D06.5-16	D-Ungstein (2006)	MDR1, Mrr1S632R, mfsM2pcr(-)	This work	
D06.5-16(ÄatrB)	Laboratory strain	Sensitive, HygR	This work	
D06.5-16(Ämrr1)	Laboratory strain	Sensitive, HygR	This work	
D06.5-25	D-Ungstein (2006)	Sensitive, BenR	This work	
D06.6-5	D-Dackenheim (2006)	MDR2, mfsM2seq(+), BenR	This work	
D06.6-5(ÄmfsM2)	Laboratory strain	Sensitive, BenR, HygR	This work	
D06.6-15 	D-Dackenheim (2006)	Sensitive	This work	
D06.6-17	D-Dackenheim (2006)	MDR1,Mrr1M251T, mfsM2pcr(-)	This work	
D06.6-22	D-Dackenheim (2008)	MDR1,Mrr1G620R, mfsM2pcr(-)	This work	
D06.7-27	D-Walsheim (2006)	MDR1, Mrr1V575M, ImiR	This work	
D06.7-27(ÄatrB)	Laboratory strain	Sensitive, ImiR, HygR	This work	
D06.7-27(Ämrr1)	Laboratory strain	Sensitive, ImiR, HygR	This work	
D06.7-33	D-Walsheim (2006)	MDR3, Mrr1G620R, mfsM2p(+)	This work	
D06.7-39	D-Walsheim (2006)	MDR3, Mrr1 n.t., mfsM2seq(+)	This work	
D08.2-2	D-Ruppertsberg (2008)	MDR3, Mrr1G620R, mfsM2pcr(+), BenR	This work	
D08.2-10	D-Ruppertsberg (2008)	MDR1, Mrr1M251T, mfsM2pcr(-)	This work	
D08.2-12	D-Ruppertsberg (2008)	MDR2, mfsM2pcr(+)	This work	
D08.3-17	D-Wachenheim (2008)	MDR1, Mrr1R407Q	This work	
D08.4-28	D-Bad Dürkheim (2008)	MDR1, Mrr1S611R	This work	
D08.5-12	D-Ungstein (2008)	MDR1, Mrr1G620R	This work	
D08.6-15	D-Dackenheim (2008)	MDR1, Mrr1G566Q	This work	
D08.6-26	D-Dackenheim (2008)	MDR3, Mrr1V575M,mfsM2pcr(+)	This work	
