# Supplementary material for: Fungicide-Driven Evolution and Molecular Basis of Multidrug Resistance in Field Populations of the Grey Mould Fungus Botrytis cinerea
Source: PLoS Pathog. 2009 Dec 18;5(12):e1000696. doi: 10.1371/journal.ppat.1000696 (PMC2785876; doi:10.1371/journal.ppat.1000696)
Supplement: Table S6 — Oligonucleotide primers used in this study. Introduced restriction sites are underlined. (0.08 MB RTF) [file ppat.1000696.s007.rtf]

Gene	Primer or primer pair	
mrr1-for1	AAAGCTTGTCCACGCAATTTGG	
mrr1-rev1	TATCCCGGGATATCGATGTTGAAGCATGAAAGAGTGGT	
mrr1-for2	TATCCCGGGAACAGTTTGTAGCGATGGAC	
mrr1-rev2	TATCCGCGGAATACGGGAATTGATAGACTC	
tubB-inv	AGTAGATGCCGACCGGGATC	
mrr1-atg	ATACCCGGGTCAACATCATGAATCCGAACAGTC	
mrr1-uaa	ATACAGCTGTTAAGAGCCAGTGAAATAATCTTGAAATG	
mrr1-pro1	TATGAATTCAGTTGCAACGGTCGTAAGAAGGAG	
mfsM2-pfor/ mfsM2-prev	TAGCCAATGGATCCTACG/ CGAGATGGATGCCATTTCAGAG	
mrr1_TF1-1/ mrr1_TF1-4	CCAATCATTCCCAATCATTCA/ GGATAGGGTATTGCGTAGATCG 	
mfsM2-KO	ATCTTTCCGCCGAGTTCCTCC	
mfsM2-KO1	GATCTAGACTCGGCTCCAGCGAAATTAC	
mfsM2-KO2	CGAATTCCCTGCTGTAGTGCTCAAACC	
mfsM2-KO3	GCTCGAGTAGATTGGCACTGGGTTC	
mfsM2-KO4	GTGGTACCTGTTCACGCGGTTAAG	
mfsM2-ATG-SmaI	ATACCCGGGCACAATGTCGGATCCTATATCAG	
mfsM2-TAG-EcoRI	ACCGAATTCCTAAGCACCACCCATAGCTGCACTC	
mfsM2-pfor-Not	TGCGGCCGCTAGCCAATGGATCCTACG	
mfsM2-prev-Sma	CCCGGGCTGAGCATGTCAGTCCAATACATCTTC	
mfsM2for/ mfsM2rev	ATTCGGCGTCGGTCTATTTG/ CCATTCAGGAAGACGGAATTGG	
KO-Hyg1-BamHI	TGGATCCCTGCAGCTGTGGAGCCGCATTC	
oliC-Sma-Rev	CTGACATCCCGGGGGATCGATTGTGATGTGATGGAG	
niaDTerm-for-Eco	CGGCGAATTCGAGGTTTTAAGTAACTGAGAGGTG	
niaDTerm-rev-Hind	GCCGCAAGCTTCAGATAGATACAGGCATTGG	
35S-gus-for-Sma	TGCCCGGGATGTTACGTCCTGTAGAAACC	
35S-gus-rev-Eco	GCGAATTCTAGAGCCAGGAGAGTTGTTG	
atrBfor / atrBrev	GCACTTGTGGCGAGTATCTATC/ TGCATCCCTCCATCCATAGC	
atrAfor/ atrArev	TGGTATTATCGCTGCTACCC/ GCAATTTGTCGTCGCATCTG	
atrDfor/ atrDrev	CGCCGGAAACATAGCCAATC/ TGTGTTAGCGAGACCCGTAG	
AtrFfor/ atrFrev	AGTGACAGGGCGTATACATC/ GAATCTTCTCGTCCGTTTCC	
AtrKfor/ AtrKrev	AATGGTAGCAGCTCTCACTCC/ CACCGATGAGACGAGTAAAGG	
BMR3for/ BMR3rev	GGATGCTGAAACTGCTGGTAAC/ AGATTACTTGACGACCGGAGAG	
BC218for/ BC218rev	CGAAAACGAAATGGAAGAGA/  ATTCCATTCCATTCCAACCA	
BC274for/ BC274rev	AGGAGACAGGCGAGATCGTA/ GGCTGGGTGTGTACTTTGGT	
BC294-2for/ BC294-2rev	 GCAAGGCGTTTTGAGCTTAC/ CTCAGCAATCAACCCAACCT	
BC63-17for/ BC63-17rev	TGCATGTTCAAGCTTTCCAG/ TCACGCCAGGTACGAGTATG	
BC302for/ BC302rev	TTTGCTTCTCTTTTAGATGATTGA/ CCCCAAACCCAAGAGATACA	
BC13-116for/ BC13-116rev	GCCAATTGCAACGATCAG/ CATCTTCCCGACTACCTC	
BC13-91for/ BC13-91rev	CACCCGTGTTCGAAGACTTTG/ CGTCACTGAAGAGGCGATTAC	
